# Dynamic of Mutational Events in Variable Number Tandem Repeats of *Escherichia coli* O157:H7

**DOI:** 10.1155/2013/390354

**Published:** 2013-09-05

**Authors:** A. V. Bustamante, A. M. Sanso, D. O. Segura, A. E. Parma, P. M. A. Lucchesi

**Affiliations:** ^1^Laboratorio de Inmunoquímica y Biotecnología, Centro de Investigación Veterinaria de Tandil (CIVETAN), Facultad de Ciencias Veterinarias, UNCPBA, Campus Universitario, Paraje Arroyo Seco S/N, 7000 Tandil, Argentina; ^2^CONICET, Argentina

## Abstract

VNTRs regions have been successfully used for bacterial subtyping; however, the hypervariability in VNTR loci is problematic when trying to predict the relationships among isolates. Since few studies have examined the mutation rate of these markers, our aim was to estimate mutation rates of VNTRs specific for verotoxigenic *E. coli* O157:H7. The knowledge of VNTR mutational rates and the factors affecting them would make MLVA more effective for epidemiological or microbial forensic investigations. For this purpose, we analyzed nine loci performing parallel, serial passage experiments (PSPEs) on 9 O157:H7 strains. The combined 9 PSPE population rates for the 8 mutating loci ranged from 4.4 × 10^−05^ to 1.8 × 10^−03^ mutations/generation, and the combined 8-loci mutation rate was of 2.5 × 10^−03^ mutations/generation. Mutations involved complete repeat units, with only one point mutation detected. A similar proportion between single and multiple repeat changes was detected. Of the 56 repeat mutations, 59% were insertions and 41% were deletions, and 72% of the mutation events corresponded to O157-10 locus. For alleles with up to 13 UR, a constant and low mutation rate was observed; meanwhile longer alleles were associated with higher and variable mutation rates. Our results are useful to interpret data from microevolution and population epidemiology studies and particularly point out that the inclusion or not of O157-10 locus or, alternatively, a differential weighting data according to the mutation rates of loci must be evaluated in relation with the objectives of the proposed study.

## 1. Introduction

Repetitive DNA, which occurs in large quantities in eukaryotic cells, has been increasingly identified in prokaryotes [1]. Repeats organized in tandem, representing a single locus and showing interindividual unit number variability, are designated variable-number tandem repeat (VNTR) loci [2]. Repeat copy number variation at these loci is the consequence of mutations resulting in the gain or loss of a certain number of tandem repeats (TRs) and can lead to a very large number of alleles [1]. These mutations are thought to occur via an intramolecular slipped-strand mispairing (SSM), which may occur in combination with inadequate DNA mismatch repair pathways [3]. Recombination may also play a role, especially in mutations involving large numbers of repeat units [4], although SSM is thought to be the predominant mutational mechanism [5, 6].

The VNTR regions have been successfully used for subtyping purposes [1, 7], and multiple-locus VNTR analysis (MLVA) involves determination of the number of repeat copy units in multiple loci [8]. Molecular epidemiology, the integration of molecular typing and conventional epidemiological studies, likely adds significant value to analyses of infections caused by pathogenic bacteria [9]. MLVA has led to the development of a highly effective typing system for use in molecular epidemiology and forensic analyses [10, 11]. In recent emergent pathogens, TRs are a source of very informative markers for strain genotyping [12, 13]. However, any change in the rate of hypervariability in these TRs is problematic when trying to predict the phylogenetic relationships among isolates [14]. Noller et al. [15] observed single locus variants during some outbreaks, and, therefore, one potential concern is that some VNTRs evolve so rapidly that multiple MLVA profiles would emerge during an outbreak initially caused by a single clone. In relation to it, Hyytiä-Trees et al. [8] proposed that during an outbreak two isolates differing in one repeat unit at one or two loci could be considered as related isolates. This is supported by the observation that isolates with five or more repeat differences at one or more loci were epidemiologically unrelated. Therefore, care has to be taken to select those repeat regions that show adequate stability over the timeframe in which the epidemiological investigations take place [16]. 

The necessity of more information on mutations and their rates in different loci to develop appropriate models to interpret MLVA data has been pointed out in several studies [8, 17–19]. Understanding of VNTR mutational rates and the factors affecting them would make MLVA more effective for epidemiological or microbial forensic investigations. Mutation rate data are valuable resources for probabilistic modeling of genetic relatedness, assessing the significance of finding genotype matches, near-matches, and mismatches [18, 20]. This information can be useful to determine the source of an outbreak, to asses true versus fortuitous disease clusters and to identify sites of exposure for human infection, among other purposes [20, 21]. 

In the present work our aim was to estimate mutation rates of *Escherichia coli* O157:H7 specific VNTR loci. For this purpose, we analyzed nine loci performing parallel, serial passage experiments (PSPEs) on nine verotoxigenic *E. coli* (VTEC) O157:H7 strains.

## 2. Materials and Methods

### 2.1. Strains

VTEC strains used in this study are listed in Table 1. The strains belong to O157:H7 serotype and were selected from those previously studied by Bustamante et al. [24] because each one of them represents a unique MLVA profile. Strains were stored at −80°C.

### 2.2. PSPE and Detection of Mutations

Parallel, serial passage experiments (PSPEs) have been previously described for *Yersinia pestis *[25] and for *Escherichia coli* O157:H7 [20]. We performed similar PSPEs on 9 *E. coli *O157:H7 strains (Table 1), generating a set of *in vitro* populations representing ~211.500 total generations (23.5 generations/colony × 100 lineages × 10 passages × 9 PSPEs). In our work, each PSPE consisted of ~100 independent clonal lineages that were each serially passaged 10 times. For each PSPE, a single isolated colony of each strain (*T* = 0) was used to start ~100 independent clonal lineages by streaking for single colonies on LB agar plates. All cultures were grown at 37°C for 24 h before the next passage. Each lineage was then serially passaged 10 times by streaking from a single colony from the previous passage. DNA was extracted from all ~100 lineages at *T* = 10 (day 10) in the 9 PSPEs by lysis of a single colony. Mutational events for each strain were visualized by MLVA, as previously described by Bustamante et al. [24]. Each lineage was tested by MLVA before and after all the passages. In order to know if the mutations were due to changes in repeat copy number or to point mutation events, the mutational products and parental alleles were sequenced. Mutational events and products for the nine strains were determined from an analysis of the *T* = 10 populations.

### 2.3. Mutation Rate Calculations and Genetic Diversity Analysis after the PSPE

Single-locus and combined 9-loci mutation rates (*μ*) were estimated for each strain by dividing the observed number of mutations by the number of total generations (GT) [20]. GT was calculated as the average number of generations per colony × the number of lineages with usable data (*n*) × the number of transfers. The number of generations per colony was determined to be 23.5 using an average of viable plate counts. In the combined single locus mutation rates, *n* was based on an average of *n* across all nine strains for each locus. To calculate the mutation rate in the combined 9 loci, *n* was based on an average of *n* across all 9 loci for each individual strain calculation and an average of *n* across all 9 loci and all nine strains for the combined strain calculations.

In order to analyze the relationship between mutation rate and repeat number, progressive linear regression analyses (PROC REG, Statistical Analysis System, Version 9.2) were performed to detect the range of repeat numbers where the slope of the regression line was not significantly different from zero.

A dendrogram showing the genetic variability cumulated during the PSPE, taking into account each strain at *T* = 0 with its derived mutated lineages, was constructed by UPGMA clustering using START version 1.0.5 [26].

## 3. Results and Discussion

Mutation is the major mechanism for generating diversity in clonal organisms, and VNTR loci are ones of the fastest mutation sequences that allow detecting genetic differences. In this study, we report the mutation dynamics of nine VNTR loci used for VTEC O157 subtyping. Eight of the nine VNTRs of this MLVA protocol have repeat units (RU) of six or seven bp and the remaining one (Vhec2), 18 bp. The genomic regions susceptible to intramolecular slipped-strand mispairing (SSM) are those that contain a short, contiguous homogenous or heterogeneous repetitive DNA sequence of 6 bp or less [1]. Repeat motifs longer than eight base pairs show typically less unit number variation but are more likely to have point mutations [1]. In this study, from the nine VNTRs analyzed, eight mutated across all PSPEs; meanwhile the Vhec2 locus did not exhibit changes in repeat number, in accordance with data from other authors [14, 17, 20]. Noller et al. [17], using a different experimental model, analyzed seven of the 9 VNTRs studied here but they only observed mutation in three VNTRs (Vhec4, O157-3, and O157-10).

In order to detect rare occurrence of mutation before *T* = 10, in some PSPEs (5 out 9) DNA was also extracted at *T* = 5 (day 5); however, we did not detect any mutations at this earlier time point.

In the nine *in vitro* PSPE populations, we observed 57 mutational events corresponding to a combined 8-loci mutation rate of 2.5 × 10^−03^ mutations/generation, and noticeably, 41 (72%) of the mutation events corresponded to locus O157-10 (Table 2). Comparison between mutated and parental allele sequences confirmed that mutations were changes involving complete repeat units, with only one exception at a single locus in one PSPE (Vhec4, FC O157) in which the change was due to a point mutation (Table 2). Of the 56 repeat mutations, 33 (59%) were insertions and 23 (41%) were deletions (Table 2). This proportion of insertion/deletion of repeats is similar to that observed by Vogler et al. [20], 62% insertions and 38% deletions, respectively. They explained the insertion bias as due to the influence of a single locus, O157-10, in a single PSPE, but in our study, on the contrary, when O157-10 was excluded from the analysis the proportion of insertions increased to 67%.

**Figure 1:**
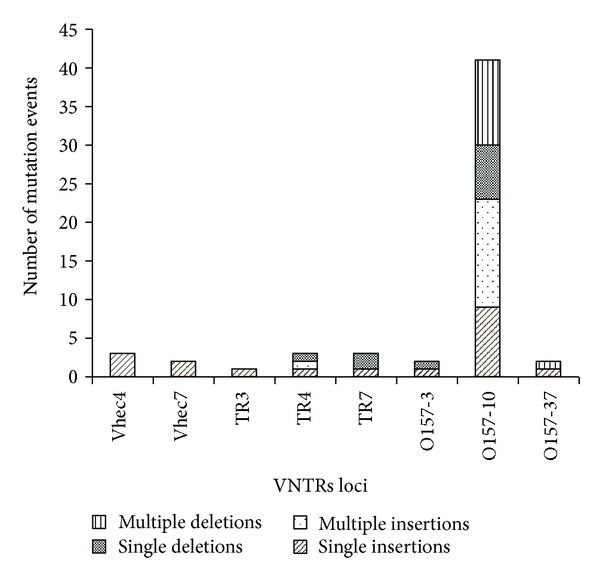
Number and kind of mutational events in each locus.

**Figure 2:**
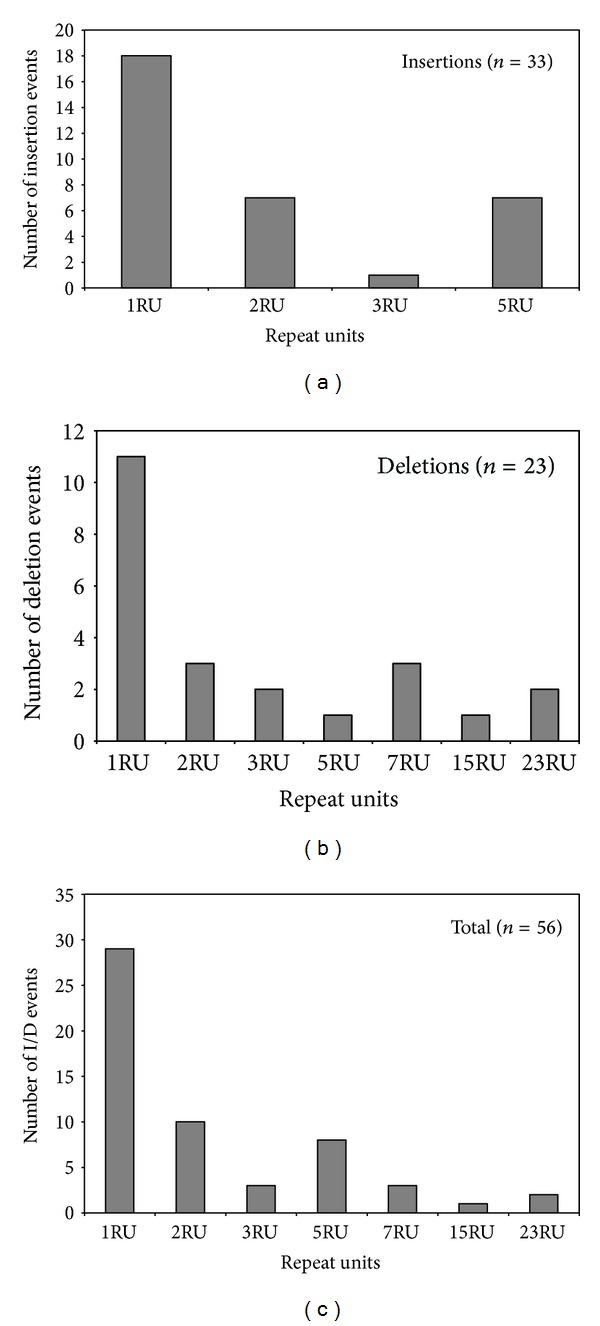
Frequency distributions of mutation products implicating complete repeat units. (a) Insertions; (b) deletions; (c) total.

Several single and multiple repeat events could be observed (Figures 1 and 2), 29 (52%) mutation events were changes due to single repeats (either insertion or deletion), and the remaining 27 (48%) involved multiple repeat changes (Table 2). This similar proportion between single and multiple repeat changes differs with one of the general properties of the mutation model for bacterial VNTRs proposed by Vogler et al. [18], who indicated that the majority of mutations involve single repeats, although a certain percentage of events consist of multiple repeats. In agreement with the results of Vogler et al. [20], the frequency of mutations followed a decreasing pattern from 1 to 3 RU (or to 5 RU if only the deletions are taken into account), and the frequency of deletions of more than 5 RU did not seem to follow any pattern (Figure 2). In the multiple repeat events the changes involved up to 23 RU, and 14 of the 27 multiple repeat mutations were “large repeat copy number” events. This last term was defined by Vogler et al. [20] in order to name mutational events involving greater than four repeat units. The occurrence of “large repeat copy number” events observed in this study (14/27 = 52%) was markedly higher than the one obtained by Vogler et al. [20] (12/47 = 26%). Interestingly, all “large repeat copy number” events detected by us were at locus O157-10, with insertions and deletions occurring at nearly the same frequency. Noticeably, two of the deletions comprised the complete VNTR; meanwhile in all the insertion events, 5 RU were inserted.

Long microsatellites are likely to mutate to shorter ones, and short microsatellites are likely to mutate to longer ones [27]. Considering the *T* = 0 MLVA profile of the different strains analyzed, the largest repeat copy numbers were present at locus O157-10, with 7 alleles comprising more than 22 RU, while in the remaining analyzed loci the largest allele was of 15 RU. VNTR alleles of very large size could be detrimental or just highly unstable, leading to the observation of a greater number of deletion events [20], but in our study, although we observed that the VNTR O157-10 was the most unstable, we did not observe a trend towards the deletions (23 insertions versus 18 deletions).

The combined 9 PSPE population rates for the 8 mutating loci ranged from 4.4 × 10^−05^ to 1.8 × 10^−03^ mutations/generation (Table 2). Our experimental design allowed us to detect mutation rates equal or higher than 4.4 × 10^−05^ mutations/generation. No mutation was observed at Vhec2 suggesting that its mutation rate is near to or less than that value, which is the detection limit given the number of generations in the PSPE population.

The mutation rates at locus O157-10 ranged from 1.3 × 10^−04^ to 4.8 × 10^−04^ mutations/generation among the nine PSPE strains (Table 2), and this locus also presented the highest mutation rate when the results of all the strains were taken into account (1.8 × 10^−03^ mutations/generation) (Table 2). Forty-one of the 57 (72%) observed mutations occurred at this locus, and when mutations associated to this locus were removed from the combined data set, the mutation rate (excluding O157-10) decreased noticeably (6.9 × 10^−04^ mutations/generation). Thus, our results, as those from Vogler et al. [20], showed that allele sizes at locus O157-10 were the most variable and therefore impacted strongly on the combined locus mutation rates.

**Figure 3:**
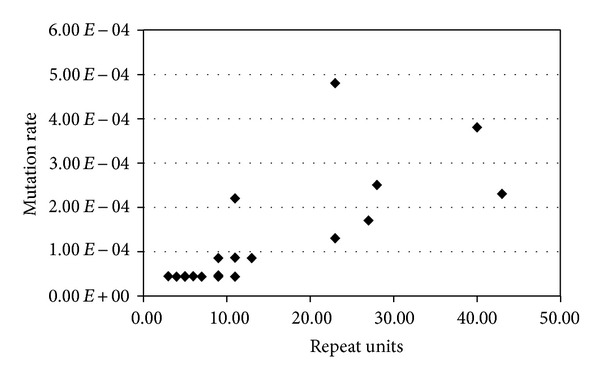
Distribution of mutation rates in relation to the number of repeats in each mutating locus. Progressive linear regression analysis (PROC REG, Statistical Analysis System, Version 9.2) showed that for alleles containing up to 13 UR the slope of the regression line was not significantly different from zero (*P* > 0.07).

It has been described in different bacterial species that mutation rates correlate with the number of repeats present in the VNTRs [18, 20, 28]. Taking into account the present results, the distribution of the mutation rates versus the number of RU present in the corresponding strain at *T* = 0 is shown in Figure 3. It can be observed that for alleles with up to 13 UR a more or less constant and low mutation rate corresponds to them; meanwhile longer alleles are associated with higher and variable mutation rates (values higher than 1 × 10^−04^). Furthermore, multiple repeat changes predominated among this second group of alleles, which correspond mainly to the locus O157-10. When we compared our results with those obtained by other authors who analysed VNTRs from VTEC O157:H7, *Yersinia pestis, *and *Vibrio parahaemolyticus* [18, 20, 28], we noticed that the distribution was similar, although they did not discuss this and instead focused on the correlation between mutation rates and repeat copy numbers. However, as O157-10 was the only locus which presented large alleles, its influence cannot be ruled out, and therefore we cannot make generalized conclusions about allele size and mutation rate.

In the present study, when results were compared among strains, strain EDL 933 noticeably showed mutations in 6 VNTRs, followed by Mat167/6 and FC O157 which presented variation in only 3 loci. On the other hand, two strains showed repeat number variation in only one locus (O157-10). Mutation rates were not so different among strains, but when they were recalculated without the inclusion of O157-10 locus, EDL 933 strain showed the highest rate followed by FC O157. On the contrary, Vogler et al. [20] did not observe a pure “strain” effect on mutation rate and indicated that instead allele size at individual loci did affect the mutation rate. Furthermore, the MLVA profile reported for the EDL 933 strain differed among several studies [8, 22, 23, 29] and also with an *in silico* analysis with the GenBank sequence (accession no. AE005174). These differences are probably due to repeated propagation of the strain and could also reflect a high mutation degree of the EDL 933 strain.

**Figure 4:**
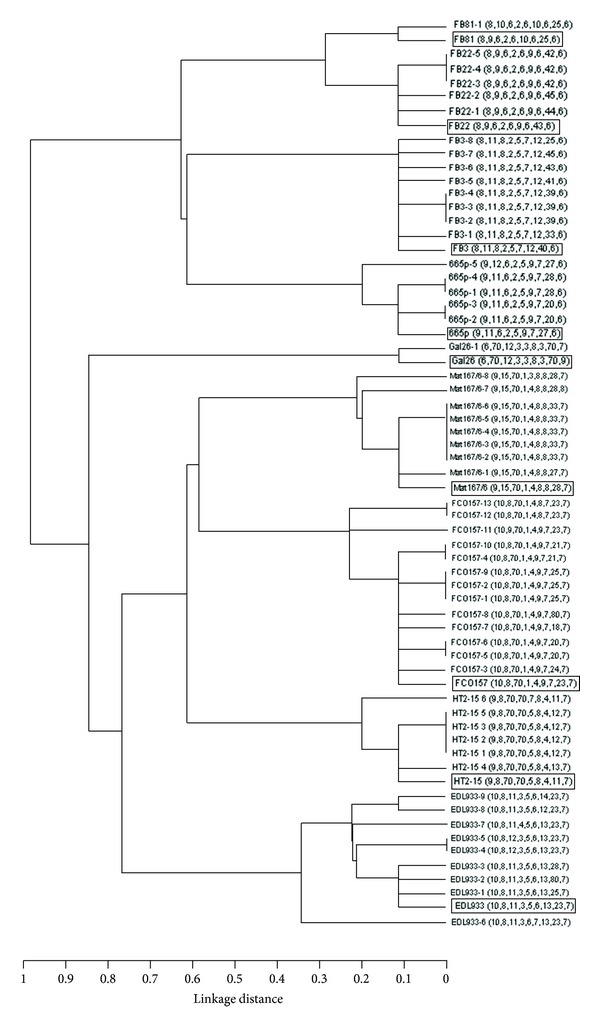
Dendrogram showing the genetic variability cumulated during the PSPE, taking into account each strain at *T* = 0 with its derived mutated lineages. String order: Vhec2, Vhec4, Vhec7, TR3, TR4, TR7, O157-3, O157-10, and O157-37. Boxed profiles correspond to the parental strains. Null alleles are indicated with 70 and with 80 when an amplification product was detected but the VNTR was missing.

MLVA is applied to study the relationships among different isolates and as an aid to determine the presence of an outbreak. The analysis of our *in vitro* PSPE population showed that each parental strain and its derived lineages became clustered evidencing their clonal relationship. The dendrogram showing the original MLVA profiles (at *T* = 0) of the strains and those of the mutated lineages is shown in Figure 4.

Cooley et al. [14] studied mutations in stressed bacteria and concluded that conditions such as elevated temperature and starvation are associated with mutations. They observed that tandem repeats were stable after limited replication, but in our study the strains were not subjected to selection pressure, and, however, we observed mutations in several VNTRs. Interestingly, their results showed that one locus (O157-10) mutated in all the conditions tested. Several studies recognized O157-10 as the most polymorphic locus [15, 19, 24, 30–33]. Furthermore, when the Pulse Net USA subtyping network recently established a standardized protocol in order to characterize verotoxigenic *Escherichia coli* O157 by MLVA, this locus was excluded because it showed too much variability during outbreaks, generating data that confounded epidemiological investigations [33]. However, Keys et al. [23] previously suggested that markers with high diversities are crucial in discriminating between closely related isolates, and they highlighted the importance of including O157-10, as an example of this kind of crucial markers. In relation with it, Urdahl et al. [19] observed that removing this locus from the analysis resulted in an MLVA that did not discriminate between closely related strains.

In summary, taking into account different points of view of several authors and our own results, care must be taken to select loci in order to be included in MLVA and to interpret data obtained from highly discriminating loci. Particularly, some aspects such as the inclusion or not of O157-10 locus in the MLVA or alternatively, a differential weighting data according to the mutation rates, have to be considered in relation with the objectives of a certain study.

## 4. Conclusions

Our set of *in vitro* populations showed that mutation rates detected for nine VNTR loci used for VTEC O157 subtyping were according to those reported previously for other bacterial VNTRs, with a similar proportion between single and multiple repeat changes. A markedly high proportion of “large repeat copy number” events was observed in this study.

Long alleles were associated with high and variable mutation rates and corresponded to locus O157-10, which presented an outstanding high mutation rate.

Our results are useful to interpret data from microevolution and population epidemiology studies and suggest, together with previously published ones, that a differential weighting of VNTR data should be applied according to the available mutation rates of loci in order to get more accurate phylogenetic relationships.

## Figures and Tables

**Table 1:** *E. coli* O157:H7 strains.

Strain	Original source	MLVA profile*
EDL 933	Reference strain	10, 8, 11, 3, 5, 6, 13, 23, 7
HT2-15	Ground beef	9, 8, 70, 70, 5, 8, 4, 11, 7
FB3	Feedlot cattle	8, 11, 8, 2, 5, 7, 12, 40, 6
FB22	Feedlot cattle	8, 9, 6, 2, 6, 9, 6, 43, 6
FB81	Feedlot cattle	8, 9, 6, 2, 6, 10, 6, 25, 6
FC O157	Feedlot cattle	10, 8, 70, 1, 4, 9, 7, 23, 7
665p	Grazing cattle	9, 11, 6, 2, 5, 9, 7, 27, 6
Gal26	Human	6, 70, 12, 3, 3, 8, 3, 70, 9
Mat 167/6	Human	9, 15, 70, 1, 4, 8, 8, 28, 7

*String order: Vhec2, Vhec4, Vhec7, TR3, TR4, TR7, O157-3, O157-10, and O157-37. The value represents the number of TRs at each locus. Vhec loci are from Lindstedt et al. [22], TR loci from Noller et al. [15], and O157-*n* loci from Keys et al. [23]. Null alleles were designated as 70.

**Table 2:** Mutation rates and products for 9 *E. coli* O157:H7 strains.

Strains and loci	RU (bp)	*n* ^a^	Total no. of mutations	Mutation rate	No. of insertions		No. of deletions	
					S^b^	M^c^	S^b^	M^c^
HT2-15								
TR4	6	99	1	4.3 × 10^−05^	0	1	0	0
O157-10	6	96	5	2.2 × 10^−04^	4	1	0	0
Total			**6**	**2.6 × **10^−04^	**6**		**0**	

EDL933								
Vhec7	7	99	2	8.6 × 10^−05^	2	0	0	0
TR3	6	97	1	4.4 × 10^−05^	1	0	0	0
TR4	6	97	1	4.4 × 10^−05^	1	0	0	0
TR7	6	97	1	4.4 × 10^−05^	1	0	0	0
O157-3	6	100	2	8.5 × 10^−05^	1	0	1	0
O157-10	6	100	3	1.3 × 10^−04^	0	2	0	1
Total			**10**	**4.3 × **10^−04^	**8**		**2**	

FB3								
O157-10	6	90	8	3.8 × 10^−04^	1	2	3	2
Total			**8**	**3.8 × **10^−04^	**3**		**5**	

Mat167/6								
TR4	6	100	1	4.3 × 10^−05^	0	0	1	0
O157-10	6	100	6	2.5 × 10^−04^	0	5	1^d^	0
O157-37	6	100	1	4.3 × 10^−05^	1	0	0	0
Total			**8**	**3.4 × **10^−04^	**6**		**2**	

Gal 26								
O157-37	6	100	1	4.3 × 10^−05^	0	0	0	1
Total			**1**	**4.3 × **10^−05^	**0**		**1**	

FC O157								
Vhec4	6	100	1^e^	4.3 × 10^−05^	0	0	0	0
TR7	6	100	2	8.5 × 10^−05^	0	0	2	0
O157-10	6	89	10	4.8 × 10^−04^	1	3	0	6
Total			**13**	**7.1 × **10^−04^	**4**		**8**	

FB22								
O157-10	6	92	5	2.3 × 10^−04^	1	1	3	0
Total			**5**	**2.3 × **10^−04^	**2**		**3**	

FB81								
Vhec4	6	92	1	4.6 × 10^−05^	1	0	0	0
Total			**1**	**4.6 × **10^−05^	**1**		**0**	

665p								
Vhec4	6	100	1	4.3 × 10^−05^	1	0	0	0
O157-10	6	100	4	1.7 × 10^−04^	2	0	0	2
Total			**5**	**2.1 × **10^−04^	**3**		**2**	

Combined data for the nine PSPEs					Insertions		Deletions	
					S^b^	M^c^	S^b^	M^c^

Vhec4		97.3	3	1.3 × 10^−04^	2	0	0	0
Vhec7		99	2	8.6 × 10^−05^	2	0	0	0
TR3		97	1	4.4 × 10^−05^	1	0	0	0
TR4		98.7	3	1.3 × 10^−04^	1	1	1	0
TR7		98.5	3	1.3 × 10^−04^	1	0	2	0
O157-3		100	2	8.5 × 10^−05^	1	0	1	0
O157-10		95.3	41	1.8 × 10^−03^	9	14	7	11
O157-37		100	2	8.5 × 10^−05^	1	0	0	1
					18	15	11	12

Total			57	2.5 ×** **10^−03^	33		23	

^a^Number of lineages with usable data.

^b^Single repeat mutation.

^c^Multiple repeat mutation.

^d^Deletion of one TR and insertion of 36 pb aleatory sequence.

^e^Point mutation.
